# The effect of meditative movement on the quality of life in patients recovering from COVID-19: A protocol for systematic review and meta-analysis

**DOI:** 10.1097/MD.0000000000023225

**Published:** 2020-11-20

**Authors:** Yanhong Wang, Gongwen Luo, Mou Shen, Xin Ge, Yanyan Huang, Tian Wei, Xianchuan Chen

**Affiliations:** a Shanghai University of Traditional Chinese Medicine Yueyang Hospital Chongming Branch of Integrated Traditional Chinese Medicine and Western Medicine; b Shanghai University of Traditional Chinese Medicine Yueyang Hospital of Integrated Traditional Chinese Medicine and Western Medicine, China.

**Keywords:** COVID-19, meditative movement, systematic review

## Abstract

**Background:**

The outbreak of a novel coronavirus (2019-nCoV)-infected pneumonia is currently ongoing all over the world. The treatment scheme is generally isolation treatment and symptomatic support treatment. While the majority of patients recover from this disease through methods above, COVID-19 Infection severely affect the physical and mental health of rehabilitation patients, as well as their living quality. Thus, meditative movement is needed to improve outcome of COVID-19 patients in recovery period.

**Methods:**

We will conduct systematic searches to identify all relevant studies without any language limitation from the following electronic databases from inception to October 2020: Medline, Ovid, PubMed, Embase, Cochrane Library, Google Scholar, China National Knowledge Infrastructure (CNKI), Chinese Scientific Journals Database (VIP), Chinese Biomedical Database, Chinese Biomedical Literature Service System and Wan fang Database. At the same time, we will search the following Clinical trial registries to identify records of on-going or completed but not yet published trials, including WHO International Clinical Trials Registry Platform (ICTRP), Trials Register of Promoting Health Interventions (TRoPHI) and Chinese Clinical Trial Registry (ChiCTR). No limits will be placed on language. The article will study the effect of meditative movement on the quality of life of convalescent patients. The main outcome will be the effect of meditative movement on the quality of life of patients in recovery period. The secondary results will select accompanying symptoms (including myalgia, cough, sputum, runny nose, pharyngalgia, anhelation, chest distress, nausea, vomiting, anorexia, diarrhea), disappearance rate, negative COVID-19 results rate on 2 consecutive occasions (not on the same day), the quality of life improved, CT image improvement, average hospitalization time, occurrence rate of common type to severe form, clinical cure rate, and mortality. Data collection and management 3 authors will independently carry out data from eligible studies in a pretested and standardized Microsoft Excel sheet, with reciprocal validation of data extraction results. Data analysis and quantitative data synthesis will be performed using RevMan software (V.5.3).

**Results:**

The findings of the study will provide new and relatively high-quality evidence in meditative movement treatment for COVID-19.

**Conclusion:**

The conclusion of systematic review will provide evidence to judge whether meditative movement is an effective intervention for patient with COVID-19 in recovery period.

**PROSPERO registration number:**

CRD42020210256.

## 1 Introduction

Coronavirus disease 2019 (COVID-19) is a novel viral illness caused by the newly emerged, highly contagious severe acute respiratory syndrome coronavirus 2 (SARS-CoV-2) in Wuhan, China, in December 2019^[1]^ Up to date (sep 25, 2020), more than 32 million confirmed cases have been reported globally, with 979.212 deaths [World Health Organization]. The most common symptoms of the patients who suffered from COVID-19 are fever, cough, fatigue, shortness of breath with abnormal chest CT, and the less symptoms are diarrhea, nausea or vomiting, nasal congestion.^[2]^ In addition, while therapies which target COVID-19 are in active development, as yet no effective treatments are available. The treatment scheme is generally isolation treatment and symptomatic support treatment.^[3]^ While the majority of patients recover from this disease through methods above, COVID-19 Infection severely affect the physical and mental health of rehabilitation patients, as well as their living quality.^[4]^ Thus, efficient, straightforward, reliable, and easily adoptable therapies are urgently needed to improve outcome of COVID-19 patients in recovery period.

Meditative movements, combining breathing, relaxation, concentration, and meditation, has been shown as a gentle movement.^[5]^ Tai Chi, yoga, baduanjin, and qigong have been regarded as the 4 classic forms of meditative movement.^[6]^ The movement combines exercise and stress reduction to engage the parasympathetic nervous system (PNS) to create a feeling of relaxation. Tai Chi (also known as Tai Chi Chuan/Quan or Tai Ji) is a form of physical activity that was originated from ancient Chinese martial art in China in the 16th century.^[7]^ The activity has been confirmed that it can improve multiple chronic diseases, such as Parkinson,^[8]^ fibromyalgia^[9]^ and chronic heart failure.^[10]^ Qigong exercise is an ancient Chinese mind-body-spirit practice taken from traditional Chinese medicine. Patients with tumors can improve their mental and physical health by exercising qigong.^[11]^ Baduanjin is a well-established treatment exercise which comprises 8 sections of gentle movements and relaxation postures. The exercise can help patients recovers from the disease.^[12]^ Yoga, which a exercise originated from ancient India since 5000 years ago, emphasizes maintenance of postures, regulation of breath, and relaxation of spirt. This exercise contributes to controlling disease and restoring health.^[13]^

Currently, there is the lack of evidence-based medicine data in the treatment of COVID-19 recovered patients, and it is important to improve negative emotions and improve quality-of- life of patients in rehabilitation.

Hence, we will conduct a systematic review and meta-analysis to answer this review question and provide alternative medical therapy for clinicians.

## 2 Methods and analysis

### 2.1 Study registration

The systematic review protocol has been in PROSPERO. Its registration number is CRD42020210256, this protocol reports consent is based on the Preferred Reporting Items for Systematic Reviews and Meta-Analyses (PRISMA) Protocols declaration guidelines.^[14]^

### 2.2 Inclusion criteria for study selection

#### 2.2.1 Type of study

We will include all papers which are related to meditative movement therapy for patients who is recovering from COVID-19. In order to ensure sufficient research objects, no matter what language the paper is written in, we will include it. But we only included randomized controlled trials (RCTs) type articles in order to evaluate results truly and objectively, Non-RCTs, quasi-randomised RCTs, randomized trials, case series, reviews, animal studies and any study with a sample size of less than 10 participants will be excluded.

#### 2.2.2 Type of participant

All COVID-19 convalescent patients, regardless of sex, age, race or educational and economic status, will be included in this review. But pregnant women, postoperative infections, psychiatric patients, patients with severe cardiovascular, and hepatorenal diseases won’t include.

#### 2.2.3 Type of intervention

In the experimental group, intervention will be exercises training such as Tai Chi or qigong or Tai Chi combined with qigong or yoga. In the Control group, intervention will included nonexercise or other physical exercise training.

#### 2.2.4 Type of outcome measures

The article will study the effect of meditative movement on the quality of life of convalescent patients. The main outcome will be the effect of meditative movement on the quality of life of patients in recovery period. The secondary results will select accompanying symptoms (including myalgia, cough, sputum, runny nose, pharyngalgia, anhelation, chest distress, nausea, vomiting, anorexia, diarrhea), disappearance rate, negative COVID-19 results rate on 2 consecutive occasions (not on the same day), the quality of life improved, CT image improvement, average hospitalization time, occurrence rate of common type to severe form, clinical cure rate, and mortality.

### 2.3 Search methods for identification of studies

We will conduct systematic searches to identify all relevant studies without any language limitation from the following electronic databases from inception to October 2020: Medline, Ovid, PubMed, Embase, Cochrane Library, Google Scholar, China National Knowledge Infrastructure (CNKI), Chinese Scientific Journals Database (VIP), Chinese Biomedical Database, Chinese Biomedical Literature Service System and Wan fang Database. At the same time, we will search the following clinical trial registries to identify records of on-going or completed but not yet published trials, including WHO International Clinical Trials Registry Platform (ICTRP), Trials Register of Promoting Health Interventions (TRoPHI) and Chinese Clinical Trial Registry (ChiCTR). No limits will be placed on language.

### 2.4 Searching other resources

#### 2.4.1 Search strategy

The retrieval strategies used in the PubMed databases are shown in Table 1. Following keywords will be comprised to develop the search strategy such as Tai chi (e.g., “Tai-ji” or “Tai-ji” or “Tai Chi Chuan”); Qigong (e.g., “Qi Gong” or “Chi Kung”); Baduanjin (e.g., “Baduanjin Qigong”, or “Baduanjin exercise”); traditional Chinese exercise; yoga; meditative movement; COVID-19(e.g., “Corona Virus Disease 2019” or “Corona Virus” or “2019-nCoV” or “SARS-CoV-2” or “2019 novel coronavirus” or “COVID-19 virus” or “coronavirus disease 2019 virus” or “Wuhan seafood market pneumonia virus”) randomized controlled trials (e.g., “randomized” or “randomly” or “clinical trial” or “random allocation” or “double-blind method” or “single-blind method”).

**Table 1:** Search strategy for the PubMed database.

Number	Search items
1	meditative movement
2	Tai Chi
3	Tai-ji
4	Tai-ji
5	Tai Chi Chuan
6	Qigong
7	Qi Gong
8	Chi Kung
9	Baduanjin
10	Baduanjin Qigong
11	Baduanjin exercise
12	traditional Chinese exercise
13	yoga
14	1 or 2–13
15	COVID-19
16	Corona Virus Disease 2019
17	Corona Virus
18	2019-nCoV
19	SARS-CoV-2
20	2019 novel coronavirus
21	COVID-19 virus
22	Coronavirus disease 2019 virus
23	Wuhan seafood market pneumonia virus’
24	15 or 16–25
25	randomized controlled trials
26	randomized
27	randomly
28	clinical trial
29	random allocation
30	double-blind method
31	single-blind method
32	27 or 28–33
33	14 and 24 and 32

### 2.5 Data collection and analysis

#### 2.5.1 Selection of studies

**Figure 1:**
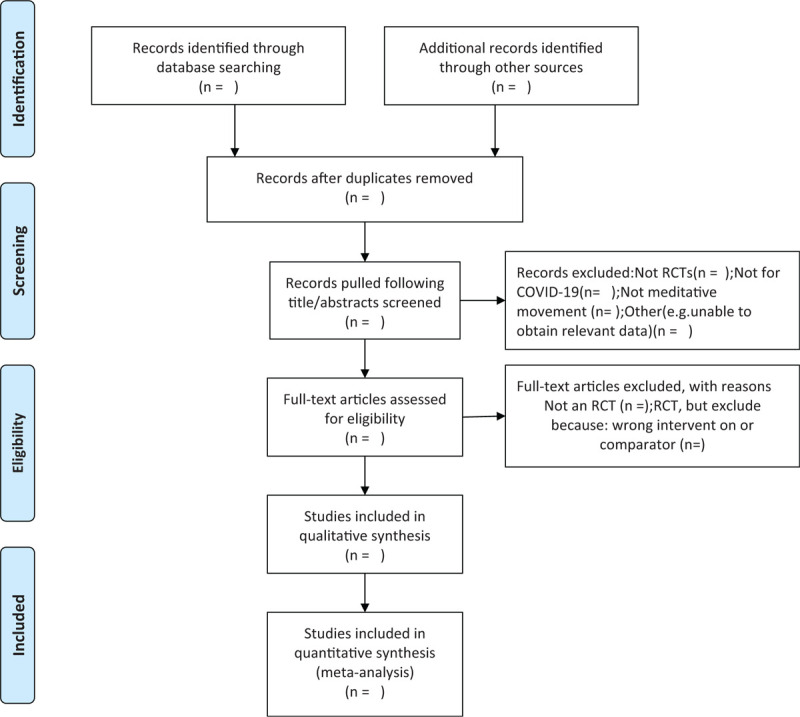
The PRISMA-P flow chart of the study selection process.

First, the whole process of article selection will be presented in a PRISMA flow chart in Figure 1. Research Selection before searching studies, investigators will discuss and decide screening criteria within the group. Three reviewers will independently screen all titles and abstracts retrieved by the systematic literature search, in order to include/exclude study. The full text of eligible studies will undergo full-text review to determine whether they satisfy the predefined inclusion criteria.

#### 2.5.2 Data extraction and management

Data collection and management 3 authors will independently carry out data from eligible studies in a pretested and standardized Microsoft Excel sheet, with reciprocal validation of data extraction results. They will then independently extract data in the following domains, such as author, published year, country, study design, sample size, general information, participants, methods, interventions, outcomes, results, adverse events, conflicts of interest, ethical approval, follow-ups, and other information. If relevant data or study information were missing, we contacted the first or corresponding author for further details. Any disagreements were resolved through discussion between the 2 authors, and further disagreements will be settled down by the third author.

#### 2.5.3 Assessment of risk of bias and reporting of study quality

We will independently assess the risk of study bias by using the Cochrane Collaboration's tool for assessing risk of bias in all Randomized Controlled Trials^[15]^

For all studies, we will assess the risk of bias from the following domains: sequence generation; allocation concealment; blinding of participants and providers; blinding of outcome assessors; incomplete outcome data; selective outcome reporting and other deviations. The risk of bias will then be divided into 3 levels: low risk, high risk and unclear. If the obtained information is insufficient or ambiguous, attempts will be made to contact the study author (s) of the study to obtain the relevant information.

#### 2.5.4 Measures of treatment effect

Data synthesis and quantitative data synthesis will be performed using Review manager software (RevMan) V.5.3. For continuous data, the mean difference (MD) or standard MD (SMD) will be used to weigh the treatment effect with 95% CIs, if there is no source of heterogeneity. If there is significant heterogeneity, the random effect model will be used to analyze data.

#### 2.5.5 Unit of analysis issues

The units of each outcome from different trials will be converted to the International System of Units before the statistical analysis.

#### 2.5.6 Management of missing data

All efforts will be made to ensure data integrity. We will contact the corresponding author if RCTs data is incomplete by making phone calls or sending emails to obtain additional data. We will exclude the incomplete or defected study if the authors are unable to be contacted. After data integrity is assured, Intention-To-Treat analysis and sensitivity analysis will be performed.

#### 2.5.7 Assessment of heterogeneity

Between-study heterogeneity will be evaluated using *I*^2^ (inconsistency index) statistic. We will consider no heterogeneity to be present the *I*^2^ test value is <50% and *P* value >1, and significant heterogeneity when the *I*^2^ test value is >50% and the *P* value is <1. When significant heterogeneity is detected, we will explore the possible causes for heterogeneity. And then a random-effects meta-analysis will be applied.

### 2.6 Assessment of reporting biases

If there are 10 or more trials included in the study, a funnel plot will be used to evaluate publication bias.

### 2.7 Data synthesis

Data analysis and quantitative data synthesis will be performed using RevMan software (V.5.3). The fixed effect model will be used for the analysis if there is no significant heterogeneity among the results (*I*^2^ ≤ 50%). And if heterogeneity is greater than 50%, the random effects model will be applied to summarize the data. If different studies exists significant heterogeneity, descriptive analysis or subgroup analysis will be conducted to explore possible reasons from both clinical and methodological perspectives.

### 2.8 Subgroup analysis

If heterogeneity is observed, subgroup analyses will be performed to investigate the potential sources. Subgroup analysis will be performed if the data are sufficient, according to different controls, interventions, and outcome measures.

### 2.9 Sensitivity analysis

If necessary, sensitivity analysis will be carried out to estimate the quality and stability of the meta-analysis results. According to the following criteria: sample size; analysis issue; methodological quality, trials with quality defects will be excluded by sensitivity analysis to make sure the quality and stability of the analysis results.

Sample size, study design, methodological quality, and missing data.

### 2.10 Grading of evidence quality

A summary of results and evidence grading will be performed using the GRADE method which is generally applied to a large amount of evidence. It will be is generally judged on the basis of risk of bias, inconsistency, indirectness, inaccuracy and publication bias. In the context of a systematic review, the ratings of the evidence reflect the extent of our confidence that reflects the true effect.^[16]^

### 2.11 Ethics and dissemination

Ethics and dissemination Ethics approval is not required in this study because no identifiable information from patients will be involved. The results of this systematic review will be presented at traditional knowledge diffusion avenues, such as international scientific meetings and publication in peer-reviewed scientific journals.

## 3 Discussion

This is the first systematic review and meta-analysis to assess effectiveness and safety of meditative movement for COVID-19 patients in recovery period. There will be 4 sections in the review: identification, study inclusion, data extraction, and data synthesis. This information will help patients and physicians make decision in treatment.

## Author contributions

**Conceptualization:** Yanhong Wang, Xianchuan Chen.

**Data curation:** Yanhong Wang, Gongwen Luo, Xin Ge, Xianchuan Chen.

**Formal analysis:** Yanhong Wang, Gongwen Luo, Xin Ge, Xianchuan Chen.

**Funding acquisition:** Yanhong Wang, Xianchuan Chen.

**Investigation:** Yanhong Wang, Mou Shen, Xianchuan Chen.

**Methodology:** Yanhong Wang, Mou Shen, Xianchuan Chen.

**Project administration:** Yanhong Wang, Mou Shen, Xianchuan Chen.

**Resources:** Yanhong Wang, Gongwen Luo, Mou Shen, Xin Ge, Xianchuan Chen.

**Software:** Yanhong Wang, Mou Shen, Xianchuan Chen.

**Supervision:** Yanhong Wang, Mou Shen, Yanyan Huang, Xianchuan Chen.

**Validation:** Yanhong Wang, Mou Shen, Xianchuan Chen.

**Visualization:** Yanhong Wang, Gongwen Luo, Xianchuan Chen.

**Writing – original draft:** Yanhong Wang, Gongwen Luo, Xin Ge, Yanyan Huang, Tian Wei, Xianchuan Chen.

**Writing – review & editing:** Yanhong Wang, Gongwen Luo, Xin Ge, Yanyan Huang, Tian Wei, Xianchuan Chen.
